# Machine learning-based estimation of trunk fat percentage and its association with cardiometabolic risk leveraging two large national cohorts

**DOI:** 10.3389/fnut.2026.1715570

**Published:** 2026-01-22

**Authors:** Liangming Zeng, Xuemin Guo, Hesen Wu, Changjing Huang

**Affiliations:** 1 Clinical Laboratory Center, Meizhou People's Hospital, Meizhou Academy of Medical Sciences, Meizhou, Guangdong, China; 2 Guangdong Engineering Technological Research Center for Clinical Molecular Diagnosis and Antibody Drugs, Meizhou, Guangdong, China; 3 Department of Cardiology, Meizhou People's Hospital, Meizhou Academy of Medical Sciences, Meizhou, Guangdong, China

**Keywords:** central adiposity, dual-energy X-ray absorptiometry, metabolic risk prediction, percent fat, trunk fat

## Abstract

**Purpose:**

This study aimed to develop and validate a machine learning model for accurate estimation of trunk fat percentage using readily available anthropometric measures, and to evaluate its discriminative performance for cardiometabolic diseases compared with conventional whole-body fat percentage.

**Methods:**

We utilized data from the National Health and Nutrition Examination Survey (NHANES; 1999–2006 and 2011–2018) as the development cohort (*n* = 30,443). Trunk fat percentage, measured by dual-energy X-ray absorptiometry (DXA), served as the gold standard. Six regression algorithms were evaluated, with model performance assessed by the coefficient of determination (R^2^). External validation was performed using the China Health and Retirement Longitudinal Study (CHARLS) cohort (*n* = 13,524), where the discriminative power for hypertension, dyslipidemia, diabetes, heart disease, and stroke was evaluated using the area under the receiver operating characteristic curve (AUC).

**Results:**

The XGBoost model demonstrated superior performance in the development cohort, achieving an *R*^2^ of 0.8509 on the test set. A simplified model utilizing only five variables (sex, waist circumference, height, weight, and age) retained 99.3% of the full model’s accuracy (*R*^2^ = 0.8450). In external validation, the machine learning-estimated trunk fat percentage consistently outperformed whole-body fat percentage across all cardiometabolic conditions, with the highest AUC improvement observed for diabetes (trunk fat AUC = 0.6607 vs. whole-body fat AUC = 0.6401; relative improvement of 3.22%). The average relative improvement in AUC across all endpoints was 2.77%.

**Conclusion:**

This study presents a highly accurate and clinically practical machine learning model for trunk fat percentage estimation using five basic anthropometric measurements. External validation confirms that trunk fat percentage is a superior biomarker for identifying cardiometabolic risks compared to whole-body fat percentage. The model provides a reliable tool for non-invasive central adiposity assessment in large-scale epidemiological studies and clinical practice.

## Introduction

Obesity represents a critical global public health challenge, with its prevalence among adults having risen markedly over the past decade (1). A key limitation in the field is the reliance on traditional measures like body mass index (BMI), which fails to distinguish between fat and lean mass or to capture the critical aspect of fat distribution (2, 3). This oversight is epitomized by the “metabolically obese, normal-weight” (MONW) phenotype, where individuals with a normal BMI exhibit metabolic abnormalities typically associated with obesity, often driven by excess central adiposity (4).

The health risks of obesity are not solely determined by the total amount of body fat but are profoundly influenced by its anatomical distribution (5). Accumulating evidence underscores that trunk (central) fat is a more potent pathogenic depot than peripheral fat, being strongly associated with insulin resistance, dyslipidemia, systemic inflammation, and an elevated risk of cardiovascular diseases (5–10). Consequently, quantifying central adiposity is essential for accurate risk stratification.

While the concept of body fat percentage (BF%) is widely accepted as superior to BMI, it still presents a limitation: it reflects a global measure that does not reveal the specific deposition of fat in the high-risk trunk region (11). This has led to growing recognition that trunk fat percentage (TF%)—the proportion of total body fat stored in the trunk—may be a more discriminative indicator of cardiometabolic risk than total adiposity alone (12–14). However, the practical assessment of body composition, especially region-specific fat distribution, remains a challenge in large-scale settings. Gold-standard techniques like dual-energy X-ray absorptiometry (DXA) are expensive and not readily available for routine use (15).

This practical constraint has spurred the development of anthropometric prediction equations as simple, cost-effective alternatives. While several models exist for estimating total body fat percentage (16–18), there is a notable gap in the literature: no universally applicable equation or machine learning model has been established specifically for predicting Trunk Fat Percentage. This gap persists despite evidence suggesting TF%'s significant clinical relevance.

Therefore, the primary aim of this study was to develop and validate a novel prediction model for TF% using advanced machine learning algorithms. Leveraging a large sample from the National Health and Nutrition Examination Survey (NHANES), we aimed to create a accurate tool based on simple anthropometric measures. Furthermore, we externally validated the model using data from the China Health and Retirement Longitudinal Study (CHARLS) and investigated the association of predicted TF% with key cardiometabolic conditions—hypertension, diabetes, dyslipidemia, heart disease, and stroke—to demonstrate its incremental utility over traditional BF% in risk assessment.

## Materials and methods

### Study population

**Figure 1:**
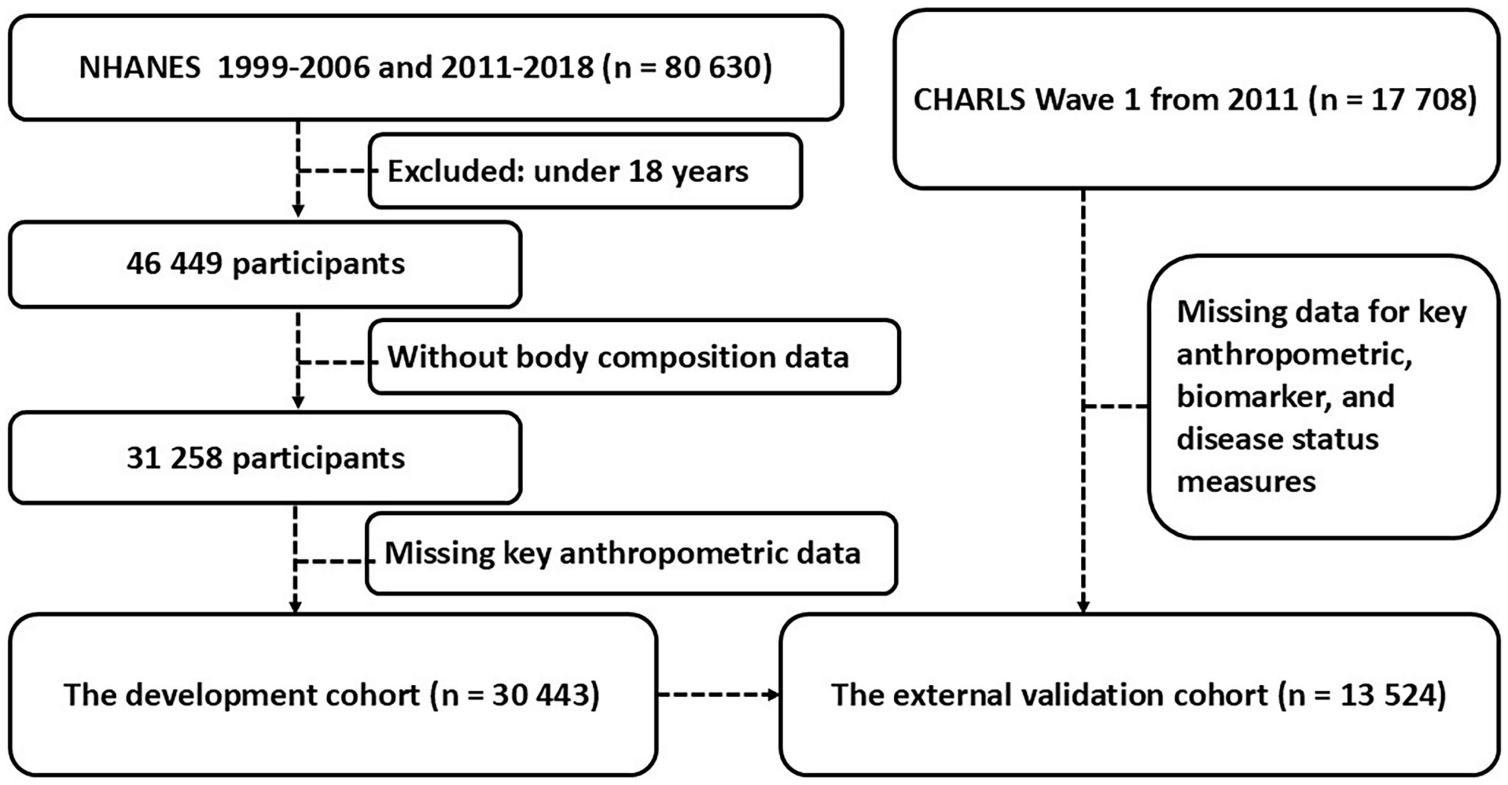
Participants’ flow chart.

In this study we utilized data from two independent surveys to develop and validate a model for estimating trunk fat percentage (Figure 1). The development cohort came from the NHANES covering 1999–2006 and 2011–2018 (19). The NHANES did not collect DXA body composition data during the 2007–2010 survey cycles; therefore, data from this period were unavailable for inclusion in the development cohort. The initial pool contained 80,630 participants. We applied sequential exclusion criteria. First, we removed individuals under 18 years of age leaving 46,449 participants. Next, we excluded those without DXA-measured body composition data resulting in 31,258 participants. Finally, we excluded individuals missing data for key anthropometric variables height weight or waist circumference which yielded our final analytical sample of 30,443 participants.

For external validation we used data from the CHARLS which initially included 17,708 participants (20). After data preprocessing and the removal of records with missing values for essential variables such as height weight and waist circumference the usable sample size was 13,524. This cohort was used to assess the model’s validity and its association with various health conditions.

### Dual-energy X-ray absorptiometry measurements

Dual-energy X-ray absorptiometry (DXA) measurements were performed by trained staff. Scans were conducted using a Hologic QDR 4500 A fan-beam X-ray bone densitometer at the Mobile Examination Center. Standard exclusion protocols were applied including recent use of radiographic contrast material or nuclear medicine tests and self-reported weight over 300 pounds or height over 6 feet 5 inches. All DXA scans underwent rigorous quality control review. Body composition metrics including lean mass fat mass and bone mineral content were derived using Hologic Discovery software version 12.1. Invalid scans were flagged as missing in the dataset. For this study the primary outcome was trunk fat percentage calculated from the DXA-derived trunk fat mass and total trunk mass.

### Anthropometric measurements and other covariates

Anthropometric data were collected by trained health technicians following standardized protocols. Height was measured with a stadiometer to the nearest 0.1 cm. Weight was measured using a calibrated digital scale to the nearest 0.1 kg. Body mass index was calculated as weight in kilograms divided by height in meters squared. Waist circumference was measured at the uppermost lateral border of the hip crest to the nearest 0.1 cm. Demographic information such as age and race/ethnicity was collected through standardized interviews.

### Biomarker and disease status collection

We analyzed laboratory biomarkers and disease status to evaluate the clinical relevance of trunk fat percentage. Biomarkers from NHANES included triglycerides total cholesterol LDL cholesterol and HDL cholesterol measured using standard laboratory methods. Fasting blood samples were used for triglyceride and LDL measurements when available. Disease status in NHANES focused on type 2 diabetes and hypertension. The CHARLS dataset provided self-reported or physician-diagnosed conditions including dyslipidemia diabetes heart disease and stroke. This approach enabled a comparative assessment of how trunk fat percentage relates to cardiometabolic risks across the two populations.

### Statistical analysis

Statistical analysis followed a structured pipeline for model development and evaluation. The NHANES development cohort was randomly split into a training set (70%) and an internal testing set (30%). Sample weights were incorporated during model training by weighting the loss function and during performance evaluation using weighted metrics (21). We evaluated six regression algorithms: Linear Regression served as the baseline model; Ridge Regression introduced L2 regularization; Lasso Regression implemented L1 regularization for feature selection; Elastic Net combined both L1 and L2 penalties (22); Random Forest provided an ensemble tree-based approach (23); and XGBoost leveraged gradient boosting (24). This suite of algorithms was chosen to cover a spectrum from interpretable linear models to complex, non-linear ensemble methods, enabling a robust comparison. All models incorporated sample weights to account for the complex survey design of NHANES.

Feature importance was determined using gain-based importance scores for tree-based models and standardized coefficients for linear models (25). We employed feature simplification strategies, including selecting top-ranked features by importance score, applying a threshold to filter out low-impact features, and prioritizing variables based on biological relevance. The performance of simplified models was rigorously assessed via cross-validation, with visual comparisons to evaluate the trade-off between simplicity and accuracy.

Subsequently, hyperparameter tuning for the top-performing models was conducted via a randomized search over a defined parameter space with 50 candidate combinations evaluated using 5-fold cross-validation (26). The final model was selected based on its performance on the internal testing set, using the coefficient of determination (R^2^) as the primary metric.

Model validation included analyzing the distribution of residuals (assessed using the Shapiro–Wilk test for normality), detecting outliers via standardized residuals, evaluating stability with learning curves, and visualizing actual versus predicted values. We also conducted subgroup analyses to assess model robustness across sex, age, and BMI categories, evaluating performance variation across these key demographic and clinical strata. Model reliability was quantified through 5-fold cross-validation, calculating the standard deviation of performance metrics to assess prediction stability.

External validation was performed using the CHARLS dataset. We compared trunk fat percentage estimates from our optimized XGBoost model against those from conventional anthropometric equations. The comparative analysis involved Receiver Operating Characteristic (ROC) curves with Area Under the Curve (AUC) evaluation for discriminating cardiometabolic conditions (hypertension, dyslipidemia, diabetes, heart disease, and stroke), correlation analysis, and distribution comparisons (20).

All analyses were performed in Python (version 3.9). Key libraries included XGBoost (v1.6.0) for model implementation, Scikit-learn (v1.0) for general machine learning utilities and evaluation, Pandas (v1.4.0) and NumPy (v1.22.0) for data manipulation, SciPy (v1.8.0) for statistical tests, and Matplotlib (v3.5.0) for visualization. Sample weights were incorporated throughout all analyses to properly represent the population-level estimates from both NHANES and CHARLS (27).

### Software implementation

We developed a publicly accessible, web-based calculator for estimating trunk fat percentage using our pre-trained XGBoost machine learning model. The application was built using the Flask framework (Python) and provides a user-friendly interface for inputting five anthropometric measurements: gender, waist circumference, height, weight, and age. This tool is designed to facilitate cardiometabolic risk assessment in clinical and research settings. The application is freely available at: https://trunkfatmodel.pythonanywhere.com/.

## Results

### Participant characteristics stratified by trunk fat percentage quartiles

The demographic and clinical characteristics of the study participants, stratified by quartiles of TF% in both the training and testing sets, are presented in Supplementary Table S1. The baseline characteristics were highly consistent between the training and testing sets, supporting the robustness of the data split.

A clear gradient was observed across TF% quartiles for most variables. Participants in higher TF% quartiles were significantly older and had higher BMI, waist circumference, and body weight (all *p*-values < 0.001). The distribution of gender was markedly different across quartiles, with a substantial majority of males in the lowest quartile (Q1: 74% in training set) and a predominance of females in the highest quartile (Q3: 82% in training set). Significant differences were also noted in the distribution of race/ethnicity across quartiles (*p* < 0.001).

Regarding cardiometabolic risk factors, the prevalence of diabetes and hypertension increased significantly with increasing TF% quartiles. Similarly, adverse lipid profiles were associated with higher TF%, including elevated levels of LDL cholesterol and total cholesterol, and lower levels of HDL cholesterol (all *p*-values < 0.001). These consistent trends in both datasets confirm that trunk fat percentage is strongly associated with known metabolic risk factors.

### Model selection and performance comparison

**Figure 2:**
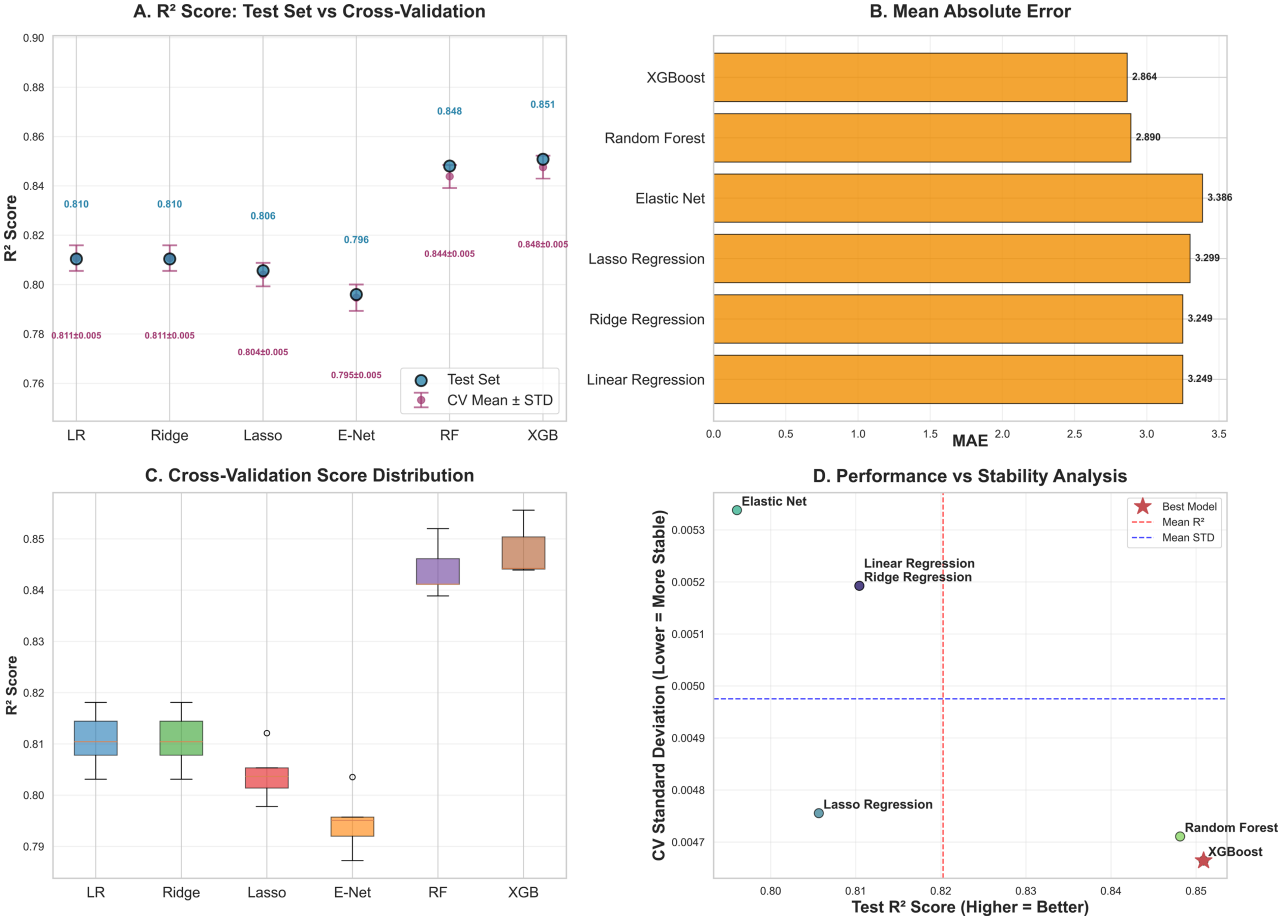
**(A)** *R*^2^ scores: test set vs. cross-validation. **(B)** Model comparison based on mean absolute error. **(C)** Cross-validation *R*^2^ score distribution and stability analysis. **(D)** Performance vs. stability trade-off across models.

The comparative performance of six algorithms for estimating trunk fat percentage is summarized in Figure 2. All models demonstrated strong predictive capability, with substantial variations in performance metrics observed across different algorithms.

XGBoost emerged as the top-performing model, achieving the highest R^2^ score on the test set (0.8509, 95% CI: 0.8439–0.8556) and exhibiting excellent cross-validation consistency (mean CV *R*^2^ = 0.8477 ± 0.0047). Random Forest closely followed with comparable performance (test *R*^2^ = 0.8481, CV *R*^2^ = 0.8439 ± 0.0047), demonstrating the superiority of ensemble tree-based methods over traditional linear models for this prediction task (Figures 2A–C).

Among linear models, Linear Regression and Ridge Regression showed identical performance (test *R*^2^ = 0.8104, CV *R*^2^ = 0.8108 ± 0.0052), suggesting minimal benefit from L2 regularization in this context, likely due to the low multicollinearity among features. The slightly lower performance of Lasso Regression (test *R*^2^ = 0.8057) and Elastic Net (test *R*^2^ = 0.7960) may be attributed to the sparsity constraint of L1 regularization, which might have excluded some predictive information, and the suboptimal balance between L1 and L2 penalties in Elastic Net. In contrast, tree-based models effectively captured non-linear relationships and interactions, resulting in superior performance.

The stability analysis revealed that tree-based models exhibited superior robustness, with Random Forest and XGBoost showing the lowest cross-validation standard deviations (±0.0047), indicating consistent performance across different data subsets (Figure 2D). This stability advantage, combined with their superior predictive accuracy, underscores the reliability of ensemble methods for trunk fat percentage estimation.

### Feature simplification and model optimization

**Figure 3:**
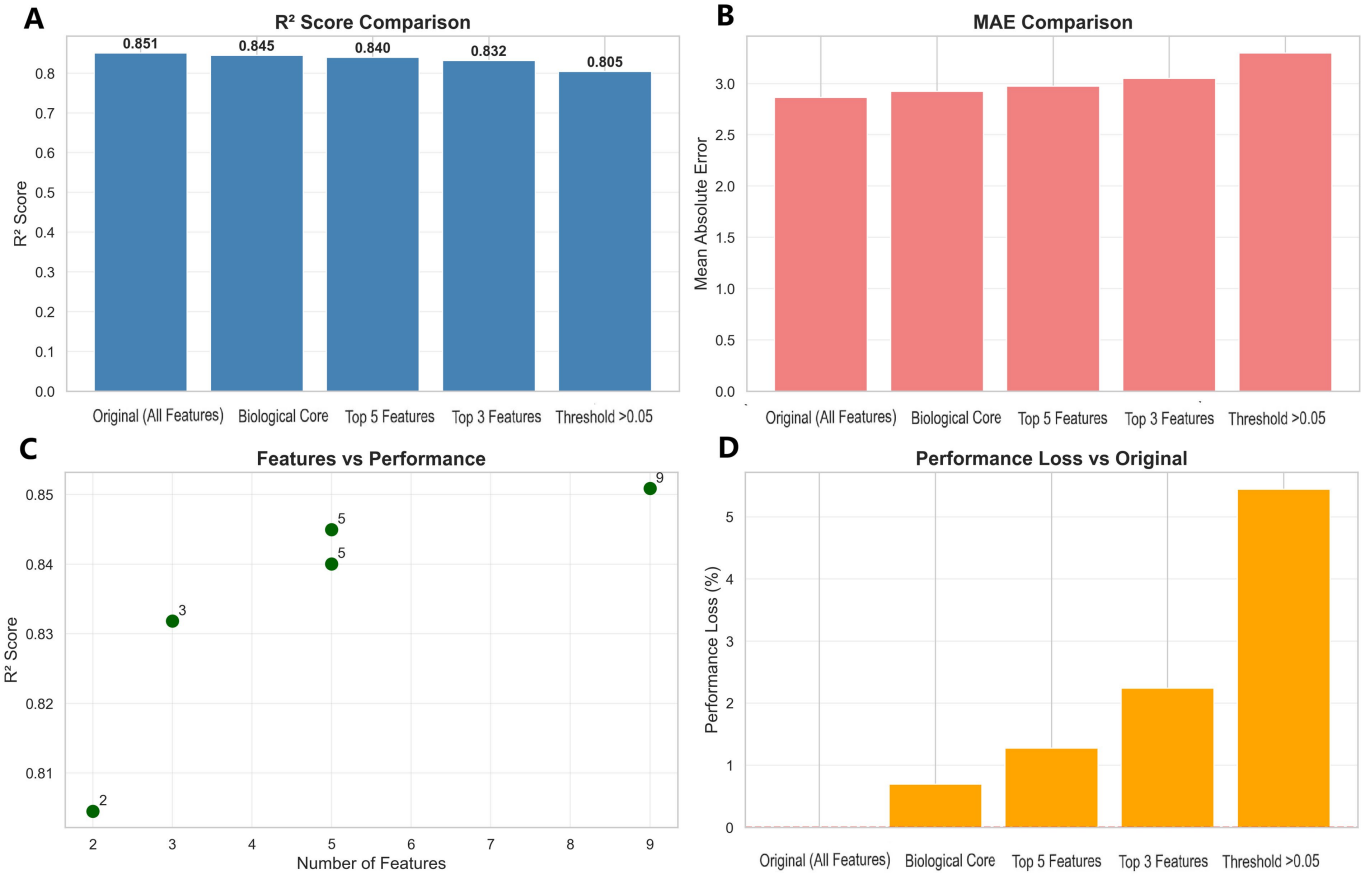
**(A)** *R*^2^ score comparison across feature selection methods. **(B)** Model MAE comparison by feature selection. **(C)** Feature quantity vs. performance relationship. **(D)** Performance loss comparison among feature selection methods.

Systematic feature evaluation revealed a minimal set of five readily available clinical measurements: sex, waist circumference, height, weight, and age. These predictors were selected based on a dual rationale: (1) their dominant collective importance in the XGBoost model (>99% of total feature importance), and (2) their universal availability in clinical and field settings. This simplified model retained 99.3% of the full model’s accuracy (*R*^2^ = 0.8450 vs. 0.8509) while reducing feature requirements by 44.4% (Figures 3A,B).

Feature importance analysis identified sex and waist circumference as the primary determinants, with anthropometric measurements (height, weight) and age providing meaningful incremental value (Supplementary Figure S1). Hyperparameter optimization yielded only marginal improvements (final *R*^2^ = 0.8471), confirming the robustness of the baseline configuration. The model demonstrated excellent cross-validation stability (*R*^2^ = 0.8434 ± 0.0047), supporting its reliability for clinical application (Supplementary Table S2).

This simplification strategy balances predictive accuracy with practical implementation needs, requiring only basic measurements routinely collected in clinical settings (Figures 3C,D). The optimized model maintains high performance while substantially reducing data collection burden, enhancing its suitability for widespread adoption in both clinical practice and epidemiological research.

### Model diagnostic analysis

**Figure 4:**
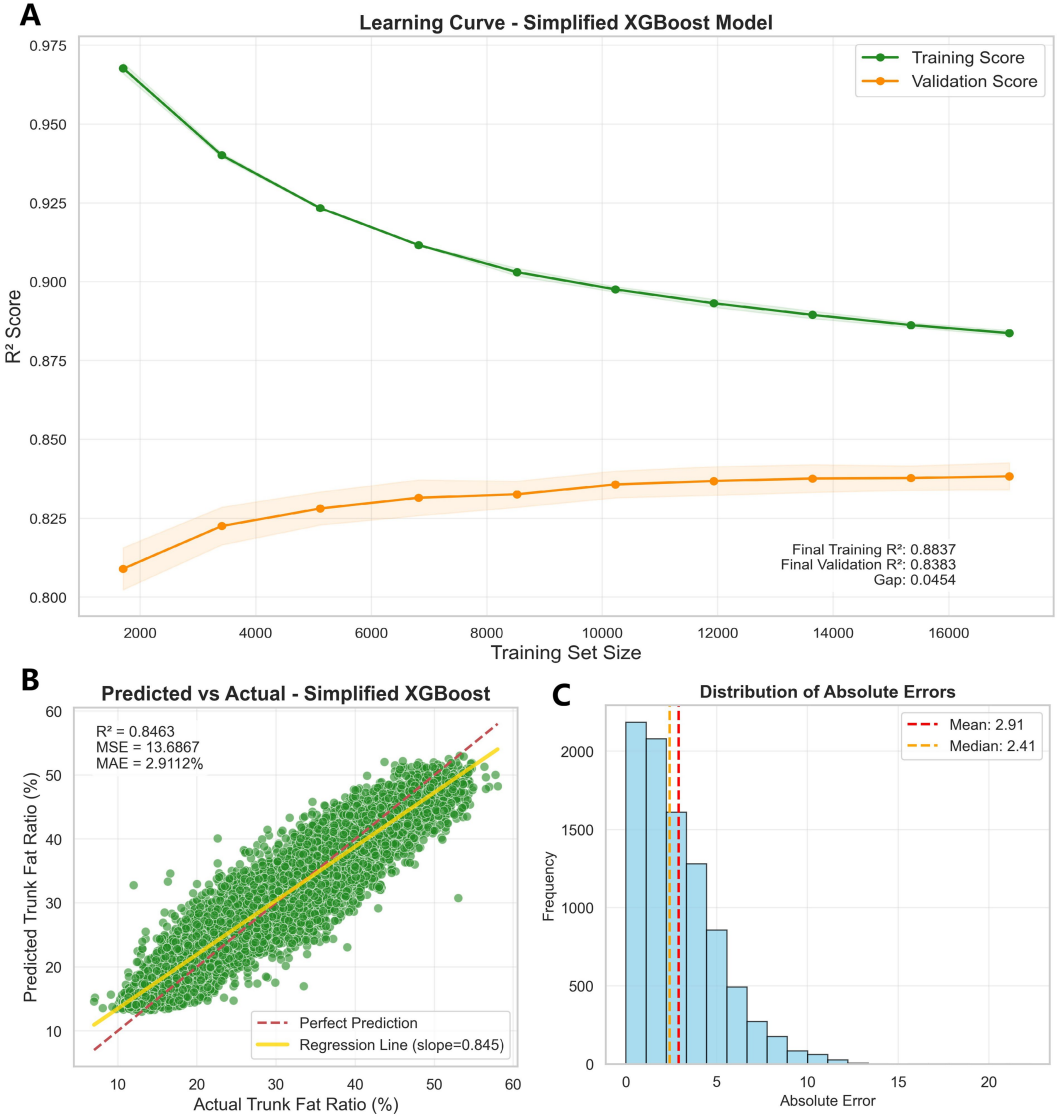
**(A)** Learning curve of the simplified XGBoost model. **(B)** Predicted vs. actual trunk fat ratio using simplified XGBoost model. **(C)** Distribution of absolute prediction errors from the simplified XGBoost model.

Comprehensive diagnostic evaluation confirmed the robustness and clinical validity of the simplified XGBoost model for trunk fat percentage estimation. The model demonstrated excellent stability across validation frameworks, with 5-fold cross-validation yielding consistent performance (*R*^2^ = 0.8423 ± 0.0049) that closely matched the holdout test set results (*R*^2^ = 0.8463), indicating minimal overfitting and strong generalizability (Figure 4B).

Residual analysis revealed a well-behaved error distribution with mean residuals approaching zero (0.025) and standard deviation of 3.70%. While formal normality testing indicated slight deviation from perfect Gaussian distribution (Shapiro–Wilk *p* < 0.001), the practical distribution characteristics remained favorable for clinical application. Outlier analysis identified only 4.9% of predictions exceeding 2 standard deviations, consistent with theoretical expectations (Supplementary Figure S2).

Learning curve analysis confirmed adequate model training, with convergence between training (*R*^2^ = 0.8837) and validation (*R*^2^ = 0.8383) performance, indicating appropriate complexity balance (Figure 4A). The diagnostic assessment supports the model’s readiness for clinical implementation, with mean absolute error of 2.91% providing practical utility for individual-level estimation while maintaining population-level accuracy suitable for epidemiological applications (Figure 4C).

### Subgroup analysis for model robustness

To evaluate the generalizability of our simplified XGBoost model, we conducted subgroup analyses across sex, age, and BMI categories (Supplementary Figure S3). The model demonstrated consistent performance across sex subgroups (male: *R*^2^ = 0.800, MAE = 2.73%; female: *R*^2^ = 0.773, MAE = 3.10%). Age-based analysis showed strong performance in individuals under 60 years (*R*^2^ = 0.854, MAE = 2.86%) and moderate performance in those 60 years or older (*R*^2^ = 0.748, MAE = 3.16%). Subgroup analyses further revealed performance variation across BMI categories, with reduced accuracy at both extremes of body composition (underweight and severe obesity), as detailed in Supplementary Figure S4. These results demonstrate that while the model maintains reasonable predictive accuracy across diverse population subgroups, performance varies across different strata, with the strongest performance observed in younger individuals (<60 years) and those in the normal or overweight category.

### External validation of disease correlation

**Figure 5:**
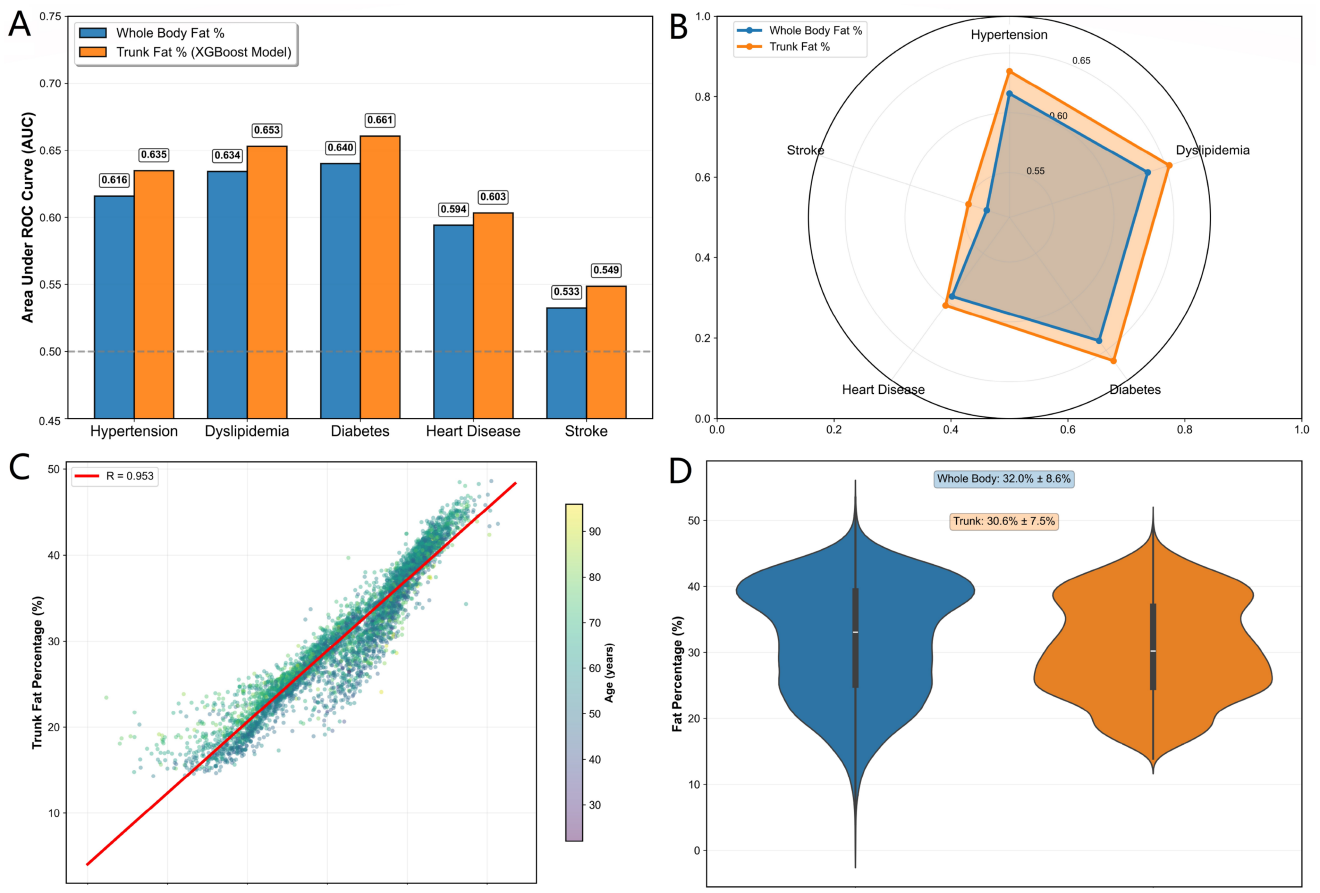
**(A)** AUC comparison between TF% and BF% for cardiometabolic diseases. **(B)** AUC radar comparison between TF% and BF% across diseases. **(C)** Correlation between TF% and BF%. **(D)** Fat distribution in TF% vs. BF%.

External validation in the CHARLS cohort (*N* = 13,044) demonstrated the superior discriminative performance of trunk fat percentage estimated by our XGBoost model compared to conventional whole-body fat percentage across multiple cardiometabolic conditions (Figures 5A,B). The machine learning-derived trunk fat percentage consistently outperformed whole-body fat percentage in predicting disease risk, with statistically significant improvements observed for all five cardiometabolic endpoints evaluated.

For diabetes risk discrimination, trunk fat percentage achieved the highest AUC improvement (AUC = 0.6607 vs. 0.6401 for whole-body fat percentage, +3.22% relative improvement). Similarly, for dyslipidemia and hypertension, trunk fat percentage showed meaningful enhancements in predictive accuracy (AUC = 0.6531 vs. 0.6342 and 0.6348 vs. 0.6160, representing 2.98 and 3.06% improvements, respectively). Even for stroke, where both measures showed limited discrimination, trunk fat percentage maintained a consistent advantage (AUC = 0.5486 vs. 0.5326).

Distribution analysis revealed strong correlation between the two fat percentage measures (*r* = 0.953), confirming that trunk fat percentage captures the essential information contained in whole-body estimates while providing incremental predictive value through its focus on central adiposity (Figures 5C,D). The external validation in this independent Chinese cohort demonstrates the generalizability of our machine learning approach and supports the utility of trunk fat percentage as a superior risk stratification tool in diverse populations.

### Clinical interpretation of TF% thresholds

To provide actionable references for cardiometabolic risk assessment, we determined the optimal TF% thresholds for predicting five key conditions in the CHARLS cohort using Youden’s index. The thresholds were 28.6% for hypertension, 28.9% for dyslipidemia, 30.8% for diabetes, 31.8% for heart disease, and 30.3% for stroke, with a median of 30.3% across conditions. These empirically derived values offer data-driven benchmarks; for instance, a TF% exceeding 28.6% suggests a higher probability of hypertension and may warrant closer monitoring. The distribution and trade-offs (sensitivity vs. specificity) of these disease-specific thresholds are detailed in Supplementary Figure S5.

## Discussion

This study successfully developed and validated a machine learning model to accurately estimate TF% using simple anthropometric measures. Our findings demonstrate that TF% is a more discriminative indicator of cardiometabolic risk than BF%. This underscores that the specific distribution of fat—rather than overall adiposity—is a critical determinant of metabolic health. The sophisticated yet practical XGBoost model effectively captured the complex, non-linear relationships underlying central adiposity.

### Trunk fat percentage as a superior risk Indicator

The strong, graded associations observed between predicted TF% and the prevalence of diabetes, hypertension, and dyslipidemia underscore the critical importance of central fat distribution. This aligns with the well-established pathophysiology linking visceral adiposity to insulin resistance and metabolic syndrome (13, 28–30). Crucially, our external validation in an independent cohort provides compelling evidence that TF% consistently outperforms BF% and BMI in predicting key cardiometabolic conditions, despite a high correlation between the two measures. The persistent, albeit modest, improvement in AUC across multiple outcomes strongly suggests that TF% captures unique pathophysiological processes related to fat distribution (31, 32), which are not represented by the global measure of total adiposity. Our results argue for a paradigm shift in risk assessment from how much fat to where the fat is located.

### Model performance and clinical translation

Our study advances the field by prioritizing extreme accessibility, in contrast to prior machine learning approaches that relied on specialized imaging or laboratory data for visceral fat assessment (33, 34). A key achievement of this study is the demonstration that machine learning (XGBoost) achieves superior predictive accuracy for trunk fat percentage compared to traditional linear models. This performance advantage indicates the presence of important non-linear relationships and interactions between anthropometric features that linear models cannot capture. Our feature importance analysis, consistent with established physiological principles, confirmed waist circumference as the most powerful predictor, followed by sex. The analysis also quantified the distinct contributions of weight, height, and age, providing both high accuracy and valuable biological insight into the determinants of central adiposity (30, 35).

A significant finding was that a parsimonious set of five core anthropometric variables proved sufficient to achieve excellent predictive performance. This favorable trade-off between simplicity and accuracy makes the model highly suitable for deployment in diverse clinical and field settings where advanced body composition assessment tools are unavailable (36). The resulting web-based tool enables identification of individuals with adverse fat distribution patterns even in the context of normal BMI, allowing for earlier and more targeted interventions in both clinical practice and population health screening (4, 37–39).

### Limitations and future directions

Several limitations should be acknowledged. While DXA-measured trunk fat percentage served as our reference standard and we adhered to NHANES quality protocols, potential calibration differences between DXA devices and across NHANES survey waves should be considered as a measurement variability source. DXA is not a direct measure of visceral adipose tissue volume, as provided by CT or MRI. The cross-sectional nature of the data establishes association but not causality; prospective studies are needed to confirm TF%'s predictive value for incident disease. Although we included major anthropometric variables, unmeasured factors such as diet, physical activity, and genetic predisposition contribute to residual variance. Furthermore, in the external validation (CHARLS), conditions such as dyslipidemia and heart disease were based on self-report, which may introduce misclassification bias and limit the achievable AUC for these endpoints. While externally validated in a distinct Asian cohort, further validation in other global populations is warranted to ensure broad applicability.

Looking ahead, several promising research directions emerge from this work. First, the model’s simplicity makes it highly suitable for integration into public health apps or portable screening devices, enabling low-cost, widespread assessment of central adiposity. Second, future studies with larger and more diverse samples could employ deep neural networks to further enhance accuracy and uncover complex, non-linear relationships between anthropometrics and trunk adiposity. Finally, while our model performed well in U. S. and Chinese cohorts, validation in other global populations (e.g., Latin American, African) is essential to confirm its generalizability and optimize its use across different ethnic and geographic settings.

## Conclusion

This study provides robust evidence that TF%, predicted with high accuracy using a machine learning model applied to simple anthropometrics, is a superior marker of cardiometabolic risk compared to total body fat percentage. The developed XGBoost model offers a powerful, accessible, and translatable tool for quantifying central adiposity, addressing a critical gap in practical risk assessment. By demonstrating that TF% provides unique predictive information beyond BF%, we underscore the paramount importance of specifically assessing central fat distribution. This model holds significant potential to enhance screening, risk stratification, and personalized management of cardiometabolic diseases.

## Data Availability

The original contributions presented in the study are included in the article/Supplementary material, further inquiries can be directed to the corresponding authors.
