# Supplementary material for: Machine learning-based estimation of trunk fat percentage and its association with cardiometabolic risk leveraging two large national cohorts
Source: Front Nutr. 2026 Jan 22;13:1715570. doi: 10.3389/fnut.2026.1715570 (PMC12872505; doi:10.3389/fnut.2026.1715570)

Supplemental Online Content

**eTable 1.** Demographic and Clinical Characteristics of the Study Population by Quartiles of Trunk Fat Percent in Training and Testing Sets

**eTable 2.** Optimized Hyperparameters and Performance Metrics of the Simplified XGBoost Model

**eTable 3.** Feature importance scores from the XGBoost model and inclusion in subsequent simplification strategies

**eFigure 1.** Feature importance analysis

**eFigure 2.** Xgboost Model Residual Analysis

**eFigure 3.** Subgroup Analysis

**eFigure 4.** Model Performance Analysis Across BMl Categories

**eFigure 5.** Clinical Thresholds Analysis

**eTable 1:** **Demographic and Clinical Characteristics of the Study Population by Quartiles of Trunk Fat Percent in Training and Testing Sets**

| **Characteristic** | **Training set** | | | |  | **Testing set** | | | | |  |
| --- | --- | --- | --- | --- | --- | --- | --- | --- | --- | --- | --- |
|  | **Overall**  N = 116,190,085^1^ | **Q1**  N = 38,607,286^1^ | **Q2**  N = 38,363,645^1^ | **Q3**  N = 39,219,154^1^ | **p-value**^2^ | **Overall**  N = 49,884,179^1^ | **Q1**  N = 16,641,181^1^ | **Q2**  N = 16,431,501^1^ | **Q3**  N = 16,811,497^1^ | **p-value**^2^ |  |
| Age in Years | 41.94± (15.13) | 36.29± (13.76) | 43.28± (14.60) | 46.18± (15.24) | <0.001 | 41.44± (15.03) | 36.10± (13.74) | 42.25± (14.37) | 45.94± (15.26) | <0.001 |  |
| Gender |  |  |  |  | <0.001 |  |  |  |  | <0.001 |  |
| Male | 10,850 (51%) | 5,370 (74%) | 4,188 (62%) | 1,292 (18%) |  | 4,662 (51%) | 2,368 (75%) | 1,755 (60%) | 539 (17%) |  |  |
| Female | 10,460 (49%) | 1,632 (26%) | 2,657 (38%) | 6,171 (82%) |  | 4,471 (49%) | 700 (25%) | 1,107 (40%) | 2,664 (83%) |  |  |
| Race/Ethnicity |  |  |  |  | <0.001 |  |  |  |  | <0.001 |  |
| Mexican American | 4,304 (8.8%) | 1,050 (6.7%) | 1,509 (10%) | 1,745 (9.5%) |  | 1,833 (8.9%) | 506 (7.8%) | 583 (9.1%) | 744 (9.7%) |  |  |
| Other Hispanic | 1,412 (6.1%) | 417 (5.5%) | 483 (6.4%) | 512 (6.4%) |  | 609 (6.1%) | 182 (5.6%) | 188 (5.8%) | 239 (7.0%) |  |  |
| Non-Hispanic White | 8,795 (67%) | 2,893 (68%) | 2,874 (67%) | 3,028 (65%) |  | 3,766 (66%) | 1,241 (66%) | 1,242 (68%) | 1,283 (66%) |  |  |
| Non-Hispanic Black | 4,716 (11%) | 1,888 (13%) | 1,194 (8.8%) | 1,634 (12%) |  | 2,004 (11%) | 781 (13%) | 499 (8.8%) | 724 (13%) |  |  |
| Other | 2,083 (6.9%) | 754 (6.5%) | 785 (7.7%) | 544 (6.5%) |  | 921 (7.1%) | 358 (7.7%) | 350 (8.5%) | 213 (5.3%) |  |  |
| Diabetes | 2,369 (8.7%) | 320 (3.3%) | 765 (8.5%) | 1,284 (14%) | <0.001 | 1,001 (8.5%) | 143 (3.5%) | 311 (8.3%) | 547 (14%) | <0.001 |  |
| Hypertension | 6,913 (30%) | 1,354 (18%) | 2,285 (31%) | 3,274 (42%) | <0.001 | 2,938 (30%) | 564 (17%) | 939 (31%) | 1,435 (42%) | <0.001 |  |
| Body Mass Index (BMI) | 28.38± (6.55) | 23.72± (3.63) | 27.89± (4.48) | 33.44± (6.88) | <0.001 | 28.38± (6.57) | 23.79± (3.61) | 27.78± (4.50) | 33.50± (6.93) | <0.001 |  |
| Waist Circumference (cm) | 96.83± (16.23) | 85.21± (10.56) | 97.67± (13.14) | 107.44± (15.99) | <0.001 | 96.62± (16.22) | 85.30± (10.35) | 97.00± (13.29) | 107.46± (16.06) | <0.001 |  |
| Body Weight (kg) | 82.22± (20.77) | 72.18± (14.85) | 82.93± (18.93) | 91.42± (22.93) | <0.001 | 82.12± (20.85) | 72.45± (14.85) | 82.51± (19.19) | 91.30± (23.12) | <0.001 |  |
| Triglycerides(mg/dL) | 300.50± (220.64) | 294.27± (241.80) | 307.83± (223.03) | 299.46± (194.76) | <0.001 | 297.83± (218.19) | 292.15± (237.12) | 306.31± (219.84) | 295.17± (195.69) | <0.001 |  |
| High-Density Lipoprotein (HDL)Cholesterol(mg/dL) | 52.46± (15.43) | 55.36± (16.09) | 50.49± (15.59) | 51.53± (14.12) | <0.001 | 52.54± (15.38) | 54.85± (15.24) | 51.10± (15.66) | 51.67± (14.99) | <0.001 |  |
| Low-Density Lipoprotein (LDL)Cholesterol(mg/dL) | 102.88± (38.75) | 94.15± (36.28) | 106.64± (39.76) | 107.80± (38.64) | <0.001 | 102.14± (37.69) | 93.09± (35.14) | 105.76± (38.12) | 107.56± (38.07) | <0.001 |  |
| Total Cholesterol (mg/dL) | 195.79± (41.65) | 184.99± (39.18) | 199.89± (42.56) | 202.42± (41.01) | <0.001 | 194.90± (39.76) | 183.60± (36.24) | 199.29± (40.38) | 201.79± (40.08) | <0.001 |  |
| ^1^Mean± (SD); N(weighted);n (unweighted) (%) | | | | | | | | | | | |
| ^2^Design-based KruskalWallis test; Pearson's X^2: Rao & Scott adjustment | | | | | | | | | | | |

**eTable 2 : Optimized Hyperparameters and Performance Metrics of the Simplified XGBoost Model**

| **Category** | **Parameter/Metric** | **Value** | **Description** |
| --- | --- | --- | --- |
| **Hyperparameters** | n_estimators | 150 | Number of boosting trees |
| **Hyperparameters** | max_depth | 6 | Maximum tree depth |
| **Hyperparameters** | learning_rate | 0.05 | Step size shrinkage |
| **Hyperparameters** | subsample | 0.9 | Subsample ratio of training instances |
| **Hyperparameters** | colsample_bytree | 1.0 | Subsample ratio of features |
| **Hyperparameters** | reg_alpha | 0.5 | L1 regularization term |
| **Hyperparameters** | reg_lambda | 2.0 | L2 regularization term |
| **Performance Metrics** | Cross-validated R² | 0.8434 | 5-fold cross-validation score |
| **Performance Metrics** | Test R² | 0.8471 | Performance on test set |
| **Performance Metrics** | MAE | 2.8958 | Mean Absolute Error |
| **Performance Metrics** | MSE | 13.6117 | Mean Squared Error |
| **Performance Metrics** | Improvement in R² | +0.26% | Compared to pre-tuning performance |

| Rank | Feature | Importance Score | Included in Simplification Strategy |
| --- | --- | --- | --- |
| 1 | Female (Sex) | 0.8320 | Biological Core, Top 5, Top 3, Threshold |
| 2 | Waist Circumference | 0.1134 | Biological Core, Top 5, Top 3, Threshold |
| 3 | Height | 0.0206 | Biological Core, Top 5, Top 3 |
| 4 | Weight | 0.0101 | Biological Core, Top 5 |
| 5 | Other Race | 0.0072 | Top 5 |
| 6 | Non-Hispanic Black | 0.0049 | – |
| 7 | Age | 0.0043 | Biological Core |
| 8 | Mexican American | 0.0040 | – |
| 9 | Other Hispanic | 0.0033 | – |

**eTable 3. Feature importance scores from the XGBoost model and inclusion in subsequent simplification strategies.**

**Performance of Simplified Models:**

- **Original Model (All 9 features):** R² = 0.8514, MAE = 2.8618
- **Biological Core (5 features: Female, Waist, Height, Weight, Age):** R² = 0.8452, MAE = 2.9203
- **Top 5 Features (by importance):** R² = 0.8399, MAE = 2.9781
- **Top 3 Features (by importance):** R² = 0.8320, MAE = 3.0459
- **Threshold (>0.05 importance):** R² = 0.8047, MAE = 3.2956

**eFigure 1:** **Feature importance analysis**

**
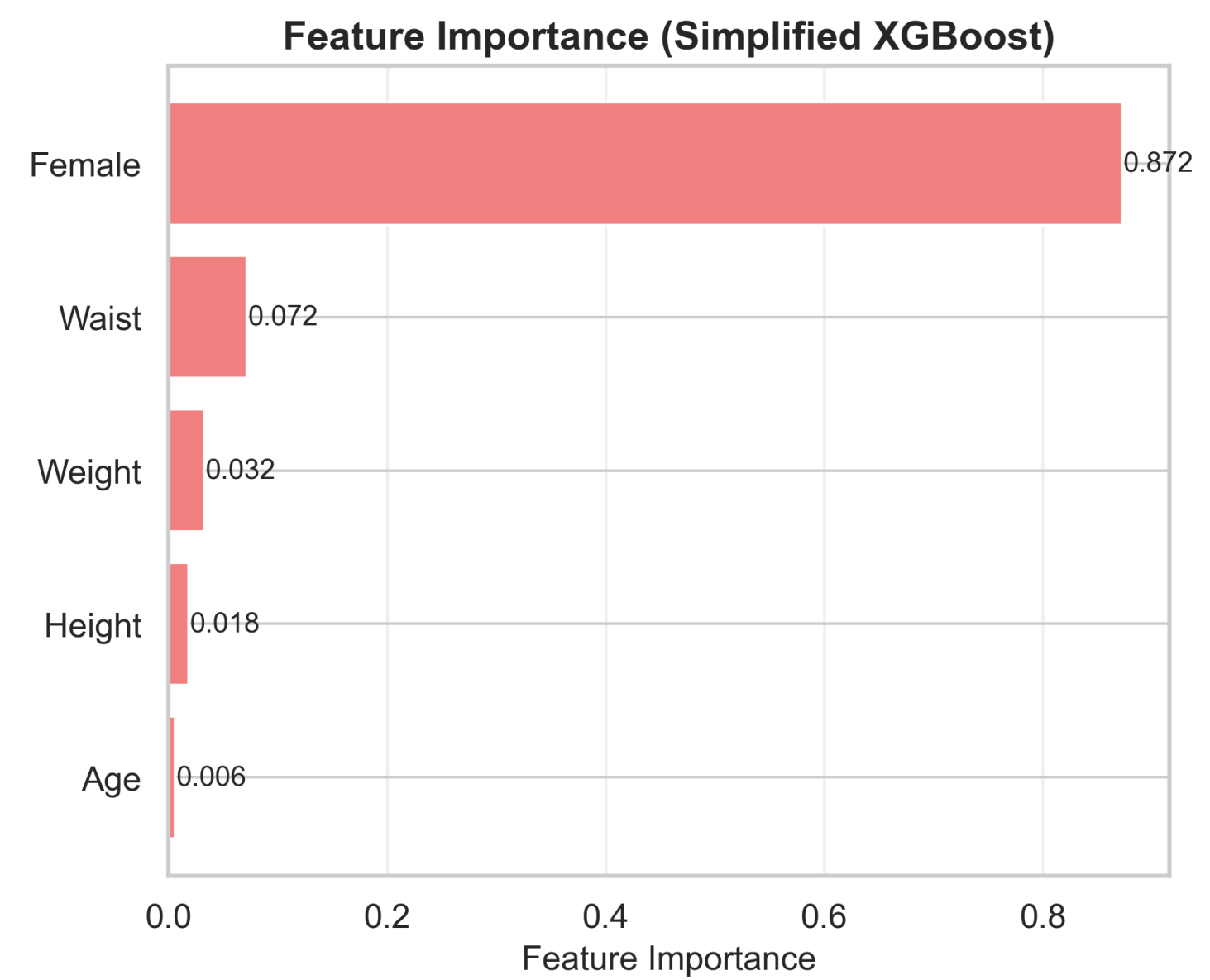
**

**eFigure 2: Xgboost Model Residual Analysis**


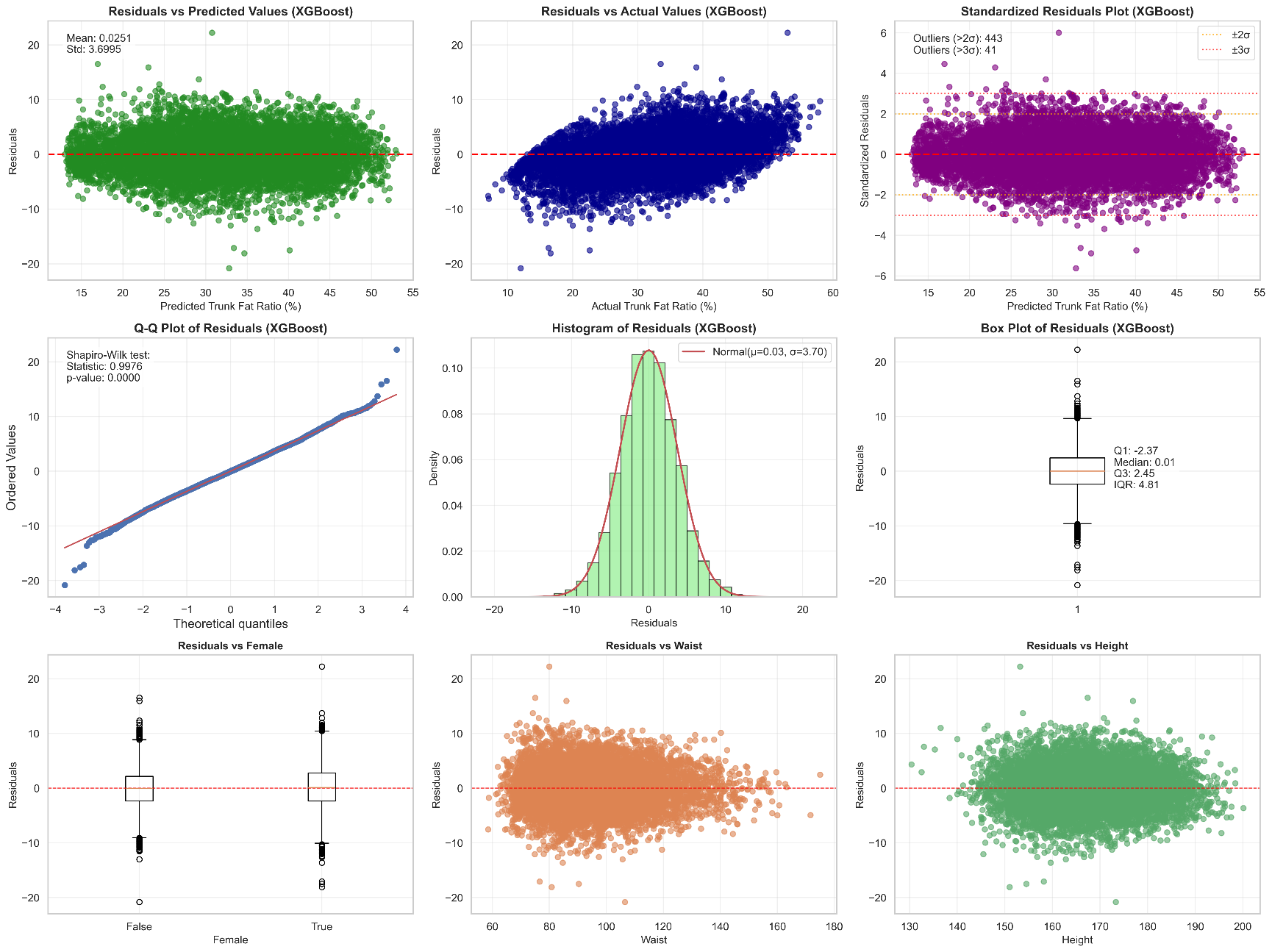


**eFigure 3: Subgroup Analysis**


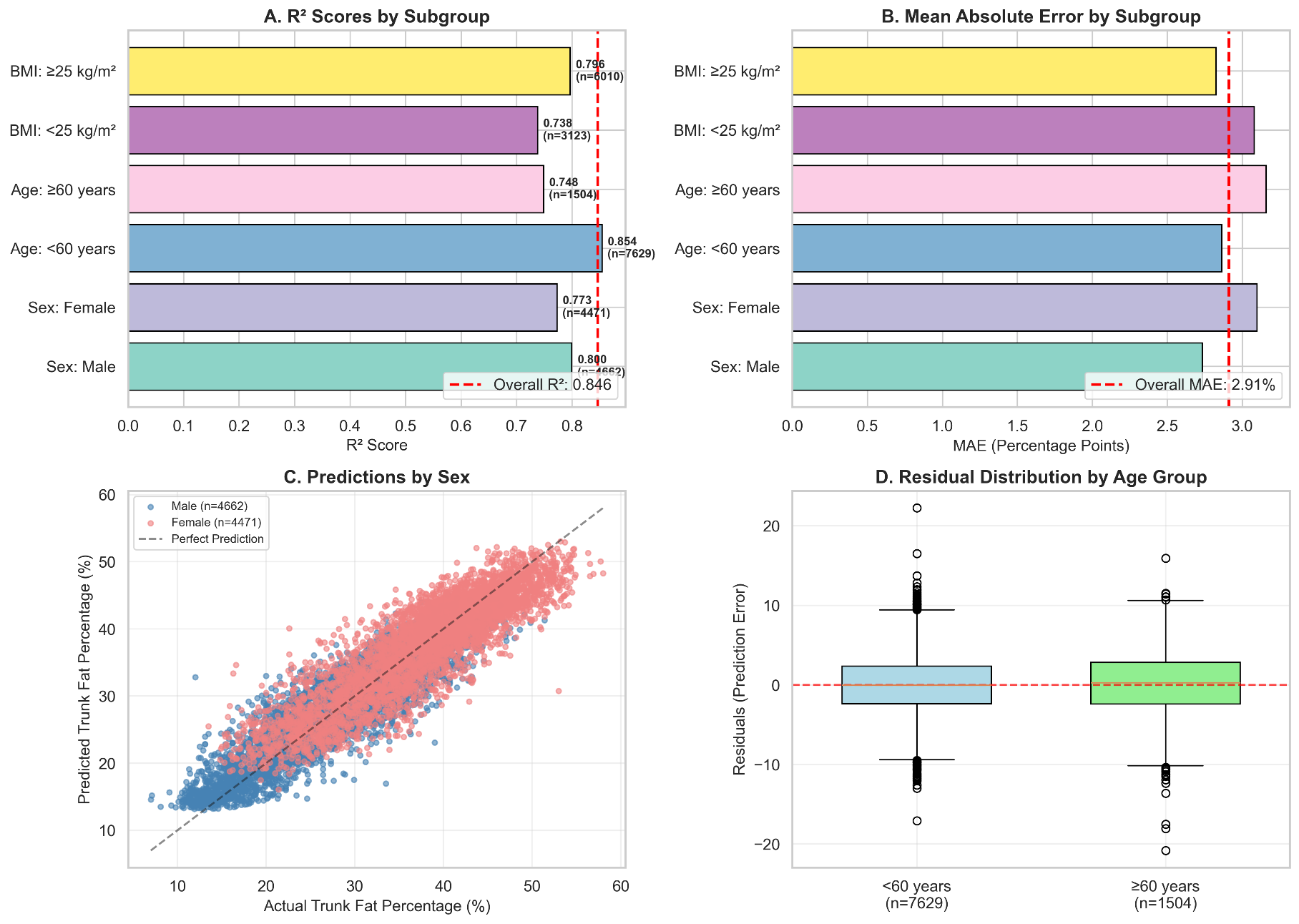


**eFigure 4: Model Performance Analysis Across BMl Categories**


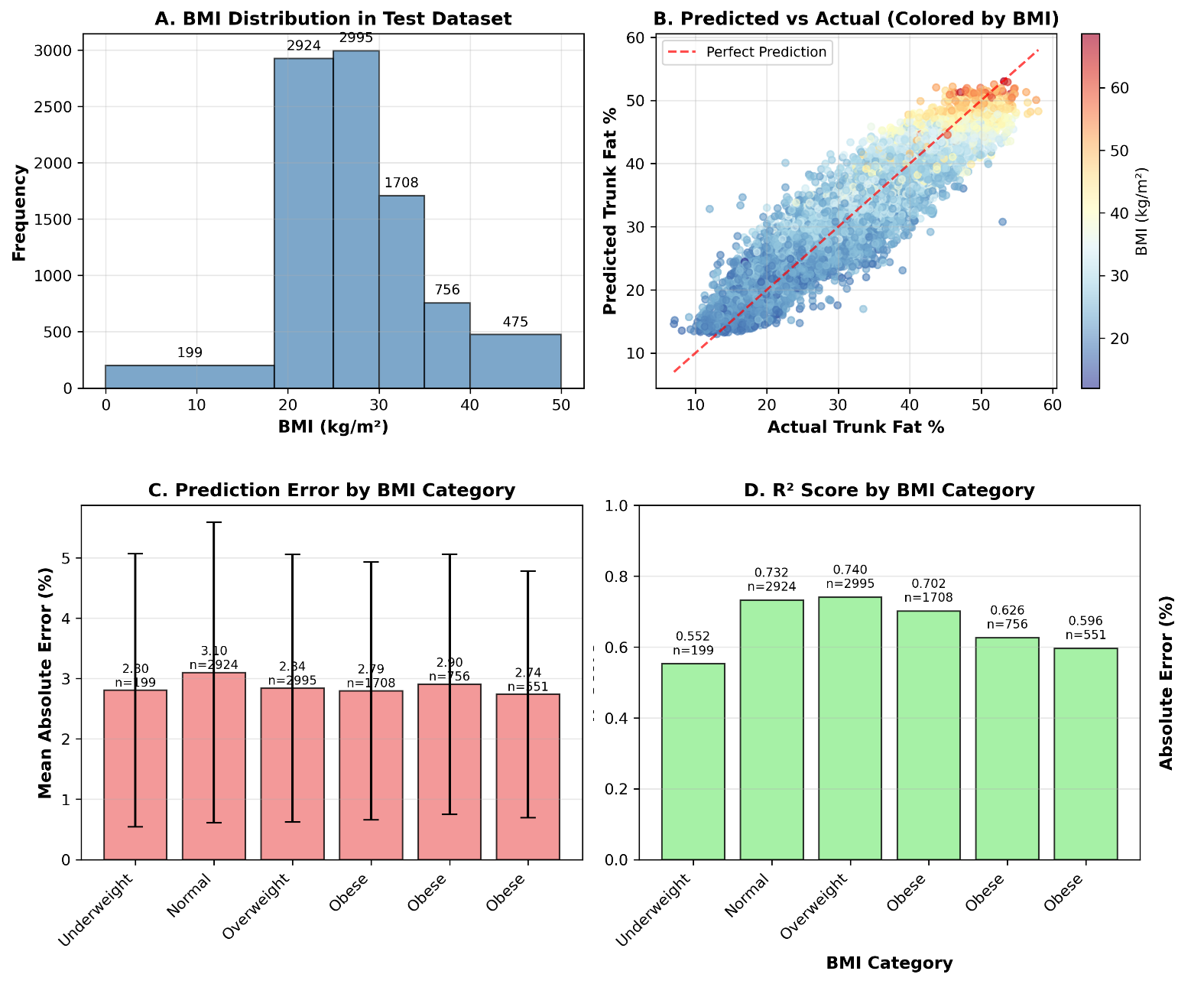


**eFigure 5: Clinical Thresholds Analysis**


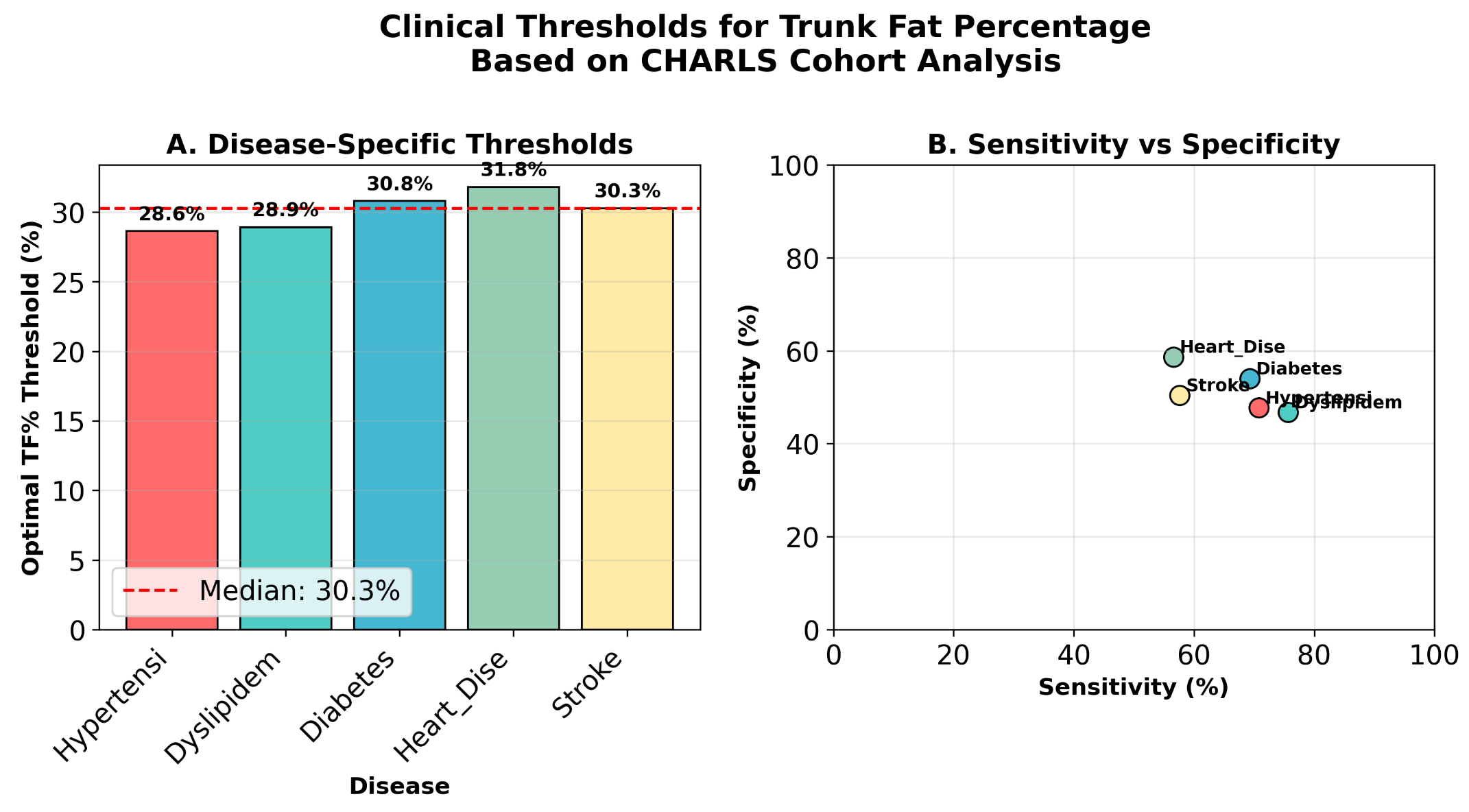

Supplement: Supplementary file 1 [file Table_1.docx]
